# Identification of Novel Immune Cell-Relevant Therapeutic Targets and Validation of Roles of TK1 in BMSCs of Systemic Lupus Erythematosus

**DOI:** 10.3389/fmolb.2022.848463

**Published:** 2022-04-11

**Authors:** Fangru Chen, Jian Meng, Wenjie Yan, Mengjiao Wang, Yunfei Jiang, Jintao Gao

**Affiliations:** ^1^ Department of Dermatology, Affiliated Hospital of Guilin Medical University, Guilin, China; ^2^ College of Biotechnology, Guilin Medical University, Guilin, China

**Keywords:** systemic lupus erythematosus, TK1, immune cells, bone marrow mesenchymal stem cells, apoptosis, cell cycle, senescence

## Abstract

**Objective:** Systemic lupus erythematosus (SLE) displays the characteristics of abnormal activity of the immune system, contributing to diverse clinical symptoms. Herein, this study was conducted for discovering novel immune cell-relevant therapeutic targets.

**Methods:** The abundance of diverse immune cells was estimated in PBMCs of SLE and healthy controls from the GSE50772 dataset with CIBERSORT approach. Immune cell-relevant co-expression modules were screened with WGCNA and relevant characteristic genes were determined with LASSO algorithm. Inflammatory chemokines were measured in serum of twenty SLE patients and twenty controls through ELISA. Bone marrow mesenchymal stem cells (BMSCs) were isolated and TK1 expression was measured in BMSCs through RT-qPCR and western blotting. TK1-overexpressed and TK-1-silenced BMSCs of SLE were conducted and apoptosis and cell cycle were measured with flow cytometry. Apoptosis-, cell cycle- and senescence-relevant proteins were tested with western blotting.

**Results:** We determined three co-expression modules strongly linked to immune cells. Five characteristic genes (CXCL1, CXCL2, CXCL8, CXCR1 and TK1) were screened and ROC curves proved the excellent diagnostic performance of this LASSO model. Inflammatory chemokines presented widespread up-regulations in serum of Systemic lupus erythematosus patients, demonstrating the activation of inflammatory response. TK1 expression was remarkably elevated in SLE BMSCs than controls. TK1 overexpression enhanced IL-1β expression, apoptosis, cell cycle arrest, and senescent phenotypes of SLE BMSCs and the opposite results were proved in TK1-silenced SLE BMSCs.

**Conclusion:** Collectively, our findings demonstrate that silencing TK1 alleviates inflammation, growth arrest and senescence in BMSCs of SLE, which highlights TK1 as a promising therapeutic target against SLE.

## Introduction

Systemic lupus erythematosus (SLE) represents an autoimmune disease with the characteristics of loss of self-tolerance and formation of nuclear autoantigen and immune complex, contributing to inflammatory response in multiple organs (Durcan et al., 2019; Kiriakidou and Ching 2020). It is prevalent among females and those with non-white races (Durcan et al., 2019; Kiriakidou and Ching 2020). SLE patients present the wide heterogeneity in clinical manifestation like rash, arthritis, and nephritis, involving one or more organs (Furie et al., 2019). Genomic analyses have enhanced our comprehending of SLE through offering crucial insights into the molecular heterogeneity of SLE. Variable genetic, hormonal, immunologic, and environmental factors result in SLE pathogenesis. However, advances in therapeutic approaches are of difficulty due to distinct biological basis as well as phenotypic presentation (Mathias and Stohl 2020). Treatment modalities like antimalarial, corticosteroid, and immunosuppressive agent remain partially effective as well as present widespread toxicity (Furie et al., 2019). Hence, it is urgently required for developing novel therapeutic agents.

SLE progression is highly complex, involving diverse cell types as well as immune and non-immune mechanisms (Furie et al., 2019). Evidences suggest the significance of innate and adaptive immune cells and inflammatory mediators in triggering and potentiating SLE (Hanaoka et al., 2020). For instance, specific regulatory T cells characterized by dichotomic immunoregulatory and T helper 17 phenotypes show elevated expression in SLE patients’ serum specimens, involving the pathogenic process of SLE (Hanaoka et al., 2020). Silencing Kv1.3 channel within T lymphocytes alleviates the clinical manifestation of SLE (Khodoun et al., 2020). To alleviate the immune imbalance of Th17 with regulatory T cell populations has become a treatment strategy against SLE (Yang et al., 2011). Despite this, SLE progression remains poorly understood. SLE bone marrow mesenchymal stem cells (BMSCs) display defects like growth arrest, senescent phenotype, secretion of cytokines as well as immunomodulation (Yuan et al., 2019). Several molecules have been found to regulate the capacity of immune modulation of SLE BMSCs. For instance, Let-7f-5p alleviates inflammation through targeting NLRP3 in BMSCs in patients with SLE (Tan et al., 2019). Moreover, silencing Let-7f in BMSCs may trigger Treg/Th17 imbalance in SLE (Geng et al., 2020). MicroRNA-663 can induce immune dysregulation via suppressing TGF-β1 production in BMSCs in patients with SLE (Geng et al., 2019). Hence, manipulating BMSCs is in favor of improving the immune response among SLE patients. Based on accumulated evidences, this study firstly characterized the landscape of immune cells among SLE with CIBERSORT algorithm. Through combining WGCNA and LASSO approaches, immune-relevant hub genes were determined and their diagnostic value was evaluated. Due to defects in immunomodulation of SLE BMSCs, we verified the expression of immune-relevant hub gene TK1 in SLE BMSCs, and investigated the effects of TK1 on BMSC growth, senescence and inflammatory response. Overall, our findings proposed the potential of TK1 as a diagnostic biomarker of SLE as well as a therapeutic target for assisting mesenchymal stem cell therapy.

## Materials and Methods

### Retrieval of gene Expression Profiling

Gene expression profiling of SLE was retrospectively gathered from the Gene Expression Omnibus (GEO) repository (https://www.ncbi.nlm.nih.gov/gds/). Two available datasets (GSE50772 and GSE81622) were finally included in our study. Microarray expression profiling of peripheral blood mononuclear cells (PBMCs) from 61 SLE patients and 20 normal donor controls was curated from the GSE50772 dataset on the Affymetrix platform (Kennedy et al., 2015). Additionally, we harvested the expression profiles of PBMCs from 15 SLE patients and 25 normal controls in the GSE81622 dataset on the Illumina platform (Zhu et al., 2016). The raw microarray data were normalized through robust multi array averaging approach. Here, GSE50772 served as the training set while GSE81622 was utilized as the testing set.

### Estimation of the Abundance of Immune Cell Populations

The cell-type identification by estimating relative subsets of RNA transcript (CIBERSORT) deconvolution algorithm (Newman et al., 2015) was adopted for computing the enrichment of infiltrating immune cell populations following the gene sets of diverse immune cell types. The degree of immune cell infiltrations was inferred utilizing the CIBERSORT package with LM22 the reference set. This analysis was implemented with 1,000 simulations as well as the results were filtered with *p* < 0.05.

### Weighted gene Co-expression Analyses

A co-expression network was established utilizing the WGCNA package (Langfelder and Horvath 2008). The RNA expression profiles and immune cell features were converted into the available format. The first 25% of genes with the highest expression variance were identified for construction of the co-expression network. Through Pearson’s correlation, the correlation coefficients between genes were computed as well as a correlation matrix was produced. Thereafter, the soft thresholding power (*β* value) was determined in accordance with the scale-free topological fit index along with mean connectivity. The optimal *β* value was confirmed through scale-free fit index = 0.9 and the largest mean connectivity through presenting gradient tests (ranging 1–20). The topological overlap matrices (TOM) were conducted through computing the topological overlapping with paired genes. Utilizing the TOM matrix, hierarchical clustering, followed by dynamic tree cut, was conducted for detecting gene modules. The smallest module size was set as 100 as well as similar modules were merged on the basis of the threshold of 0.25. For excavating the co-expression modules that presented high associations with the immune cells, correlation analyses of modules with immune cells were carried out through computing Spearman’s correlation coefficients between module eigengenes (MEs) and immune cell characteristics. The ME represents the main components of RNA expression profiles in a specific module. Modules that displayed significant interactions with immune cells were determined. Thereafter, gene significance (GS) as well as module membership (MM) were separately computed for intramodular analyses. GS represents the interaction of RNA expression with immune cell feature. Additionally, MM indicates the correlation of RNA expression profiling with ME of a specified module. The module comprising of genes with remarkable associations between GS and MM was regarded meaningful.

### Functional Enrichment Analyses

Gene ontology (GO) analyses were adopted for classifying the genes into three categories in accordance with the bio-function, comprised of biological processes (BPs), cellular components (CCs), and molecular functions (MFs). Additionally, Kyoto encyclopedia of genes and genomes (KEGG) pathway enrichment analyses were conducted for exploring the biological features. GO and KEGG pathway analyses were both implemented with the clusterProfiler package (Yu et al., 2012). Adjusted *p*-value<0.05 was regarded as significant enrichment, which was computed with the Benjamini and Hochberg approach.

### Determination of Hub Genes

Through the online database Search Tool for Retrieval of Interacting Genes/Proteins (STRING; http://string-db.org), the protein-protein interaction (PPI) network was conducted (Szklarczyk et al., 2017). The Molecular Complex Detection (MCODE) approach (Doncheva et al., 2019) was adopted for screening modules of the PPI network in accordance with node score cut-off = 0.2, degree cut-off = 2, k-core = 2 and max depth = 100.

### Least Absolute Shrinkage and Selection Operator Analyses

LASSO approach was adopted to reduce complexity as well as prevent overfitting of the model according to the optimal value of lambda based on the expression profiles of hub genes utilizing the glmnet package (Engebretsen and Bohlin 2019). The diagnostic model was conducted through combining the optimal characteristic gene expression with the regression coefficient weight computed from the multivariate model.

### Receiver Operator Characteristic Curve Analyses

The multivariate model with integrated characteristic genes were adopted for determining the high sensitivity and specificity for diagnosing SLE. The ROCs were drawn both in the GSE50772 and GSE81622 datasets as well as area under curves (AUCs) were computed to evaluate the performance of the model utilizing the pROC package (Robin et al., 2011).

### Gene Set Enrichment Analyses

GSEA was employed to investigate whether certain sets of genes displayed significant difference between two groups (Subramanian et al., 2005). The “c2. cp.kegg.v7.1. symbols” gene set from the Molecular Signatures Database (https://www.gsea-msigdb.org/gsea/msigdb) (Liberzon et al., 2015) was utilized as the reference gene set. The default settings were set with 10,000 gene set permutations and terms with nominal *p* < 0.05 were regarded as significant enrichment.

### Patients

Twenty SLE patients and twenty healthy volunteers were recruited in this project. SLE patients were diagnosed at Affiliated Hospital of Guilin Medical University in accordance with American College of Rheumatology. The inclusion criteria were as follows: 1) all patients were diagnosed as SLE; 2) the participants had a complete understanding of the content of this study; 3) the participants’ information was complete. The exclusion criteria were as follows: 1) patients with rheumatoid arthritis, cardiovascular and cerebrovascular diseases, liver and kidney failure, diabetes, mental illness, etc.; 2) females who were breastfeeding or pregnant; 3) patients who recently took drugs that had an effect on this study. Each participator provided written informed consent as well as this project was approved by the Ethical Committee of Affiliated Hospital of Guilin Medical University (YXLL-2016-WJWZC-14).

### Enzyme-Linked Immunosorbent Assay

ELISA kits of interferon-γ (IFN-γ; H025), interleukin-12 (IL-12; H010), interleukin-6 (IL-6; H007-1-1), interleukin-13 (IL-13; H011), interleukin-18 (IL-18; H015), and interleukin-1β (IL-1β; H002) were purchased from Nanjing Jiancheng Bioengineering Institute (China). Their serum levels were measured in accordance with the manufacturer’s instructions.

### Bone Marrow Mesenchymal Stem Cell Isolation and Culture

Bone marrow was harvested from iliac crest from five SLE and five controls. Thereafter, bone marrow mononuclear cells were extracted utilizing Ficoll separation medium (TBD, China), followed by resuspending in low glucose DMEM (Gibco, United States) plus 10% FBS and 1% antibiotic-antimycotic solution. All cells were maintained in an atmosphere with 5% CO_2_ at 37°C. Following 2 days, medium including non-adherent cells was exchanged every 3 days. When the confluence reached 90%, cells were digested through 0.25% trypsin-ethylenediaminetetraacetic acid. At passage four, BMSCs were collected for subsequent assays.

### Real-Time Quantitative Polymerase Chain Reaction

Total RNA was extracted from BMSCs utilizing TRIzol (Invitrogen, United States). The cDNA was retrieved through PrimeScript™ RT reagent kit with gDNA Eraser. Thereafter, RT-qPCR was conducted through SYBR^®^ Premix Ex Taq™ II and Bulk kit (Takara, China). The RT-qPCR condition was one cycle of denaturation at 95°C lasting 30 s as well as forty cycles of denaturation at 95°C lasting 5 s and annealing at 60°C lasting 34 s. GAPDH was adopted as an internal reference. The primer sequences were as follows: TK1, 5′-GGG​CAG​ATC​CAG​GTG​ATT​CTC-3’ (forward), 5′-TGT​AGC​GAG​TGT​CTT​TGG​CAT​A-3’ (reverse); GAPDH, 5′-ACA​ACT​TTG​GTA​TCG​TGG​AAG​G-3’ (forward), 5′-GCC​ATC​ACG​CCA​CAG​TTT​C-3’ (reverse). The 2^−ΔΔCq^ approach was adopted for quantification.

### Western Blotting

BMSCs were lysed through protein extraction reagent (Thermo Scientific, United States) and protease inhibitors. The protein was collected under centrifugation of 12,000 g lasting 15 min at 4°C. Protein concentration was measured utilizing NanoDrop 2000 spectrophotometer. 20 µl protein was separated through 12% SDS-PAGE as well as transferred onto polyvinylidene fluoride membrane. The membrane was sealed through 5% non-fat dry milk, followed by incubation overnight at 4°C with primary antibodies targeting TK1 (1:1,000; ab239509; Abcam, United States), GAPDH (1:1,000; ab181602; Abcam, United States), Cleaved caspase-3 (1:500; ab2302; Abcam, United States), Bax (1:500; ab53154; Abcam, United States), Bcl-2 (1:1,000; ab196495; Abcam, United States), CyclinD1 (1:10,000; ab134175; Abcam, United States), p53 (1:10,000; ab32389; Abcam, United States), p21 (1:1,000; ab188224; Abcam, United States), and p27 (1:1,000; ab190851; Abcam, United States). Thereafter, the membrane was incubated by secondary antibody (1:2,000; ab7090; Abcam, United States) lasting 2 hours at room temperature. The immunoreactive protein was visualized through a chemiluminescence kit (Beyotime, China) as well as band density analyses were conducted utilizing ImageJ software.

### Transfection

For generating TK1-overexpressing BMSCs, the cDNA of TK1 was PCR-amplified as well as cloned into the EcoRI and XbaI sites of the LV-003 lentivirus vectors (GenePharma, China). The lentivirus vectors were co-transfected by packaging vectors into 293T cells for producing recombinant lentivirus. Thereafter, BMSCs were co-cultured with recombinant lentivirus in the culture medium containing 2 μg/ml puromycin for generation of TK1-overexpressing BMSCs as well as empty vector BMSCs. Si-TK1 was acquired from GenePharma company (China). BMSCs were planted onto a 6-well plate. Following 12 h, 100 nM siRNA was transfected into BMSCs utilizing Lipofectamine 2000 (Invitrogen, United States) in accordance with the manufacturer’s protocol. At 48 h post-transfection, BMSC expression was measured with RT-qPCR and western blotting.

### Flow Cytometry Analyses

Apoptosis and cell cycle of BMSCs were measured with flow cytometry analyses. Following trypsinization and rinsing by PBS, BMSCs were fixed with 70% ethanol as well as incubated on the ice lasting 15 min. Thereafter, BMSCs were labeled by propidium iodide (PI)/RNase staining solution (Beyotime, China), followed by incubation at room temperature lasting 15 min. Apoptosis and cell cycle were analyzed utilizing BD FACSAria™ (BD, United States). Data analyses were conducted with FlowJo software.

### Statistical Analyses

Student’s *t* test was adopted for determining the difference between two groups. Meanwhile, one-way analyses of variance followed by post hoc Bonferroni correction were conducted for comparing multiple groups. Data are presented as mean ± SD. All statistical analyses were implemented with R software and GraphPad Prism software. The difference was regarded significant at a *p* < 0.05.

## Results

### Establishment of Immune Cell-Relevant Co-expression Modules

**FIGURE 1:**
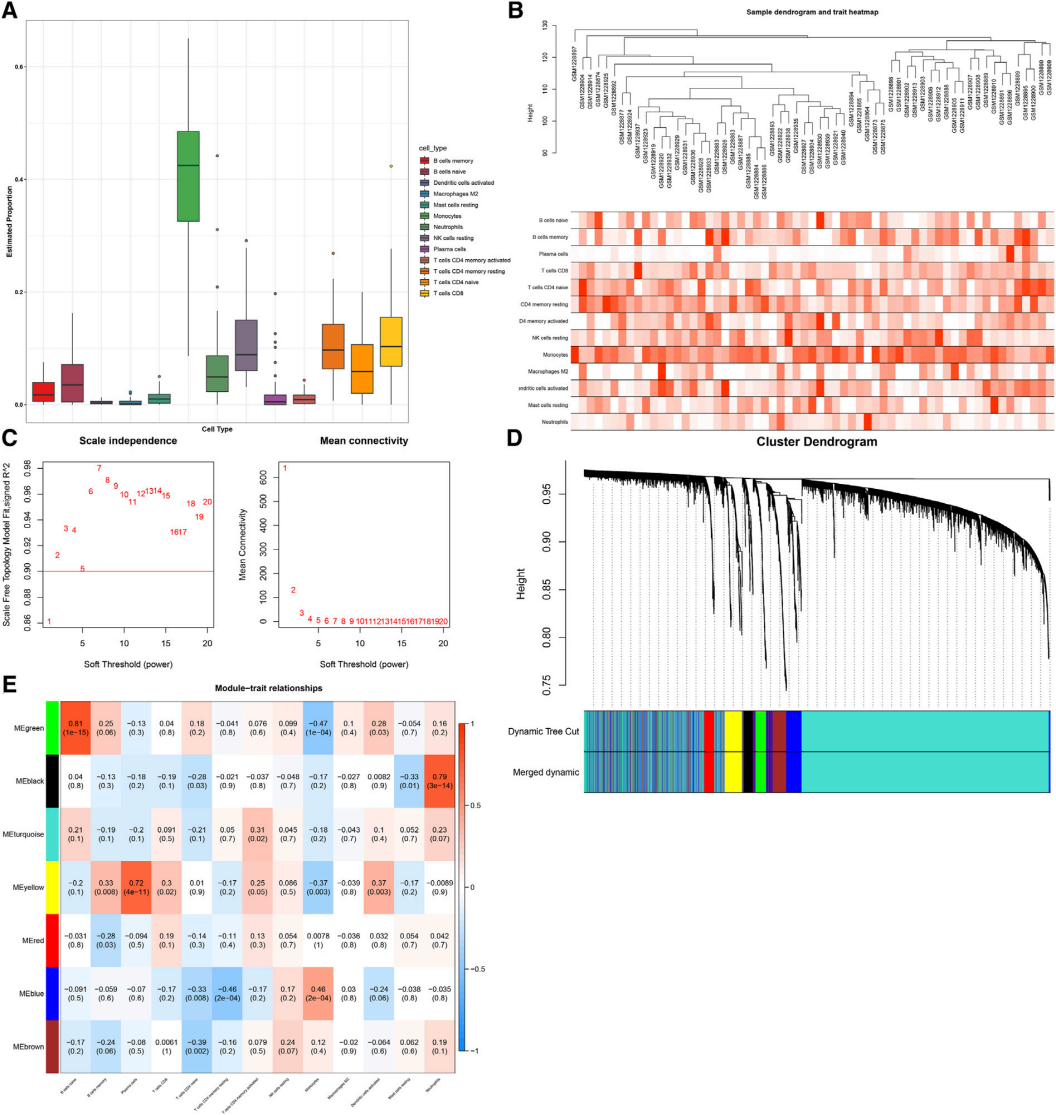
Establishment of immune cell-relevant co-expression modules with WGCNA approach. **(A)** Quantification of the abundance of diverse immune cells in SLE specimens through the CIBERSORT approach. **(B)** Sample clustering for detecting outliers. **(C)** Determining soft-thresholding power (*β*) through analyses of (left) scale-free fit index and (right) mean connectivity. **(D)** Dendrogram of consensus module eigengenes. Gene dendrogram is generated following clustering the dissimilarity. **(E)** Heatmap of the association between diverse immune cells and module eigengenes. Each row and column separately indicate a consensus module and immune cells. The cells are colored in line with the correlation (red: positive correlation; blue, negative correlation). The intensity of the color reflects the strength of the correlation.

With the CIBERSORT approach, we quantified the infiltration levels of diverse immune cells of SLE in the GSE50772 dataset, as depicted in Figure 1A. For discovering immune cell -relevant co-expression modules, we carried out WGCNA in SLE. In Figure 1B, all specimens were in the clusters and pass the cutoff thresholds. To guarantee a scale-free co-expression network, the optimal soft-thresholding *β* value was determined when scale-free fit index = 0.9 (Figure 1C). Following dynamic tree cut approach, seven co-expression modules were finally clustered (Figure 1D). Analyses of module-trait relationships demonstrated that black module displayed a strong correlation to neutrophils (*r* = 0.79; *p* = 3e-14); green module showed a strong interaction with B cells naïve (*r* = 0.81; *p* = 1e-15); and yellow module was strongly linked to plasma cells (*r* = 0.72; *p* = 4e-11), as depicted in Figure 1E. Above modules were regarded as immune cell-relevant co-expression modules.

### Analyses of Immune Cell-Infiltration-Relevant Genes and Their Biological Significance

**FIGURE 2:**
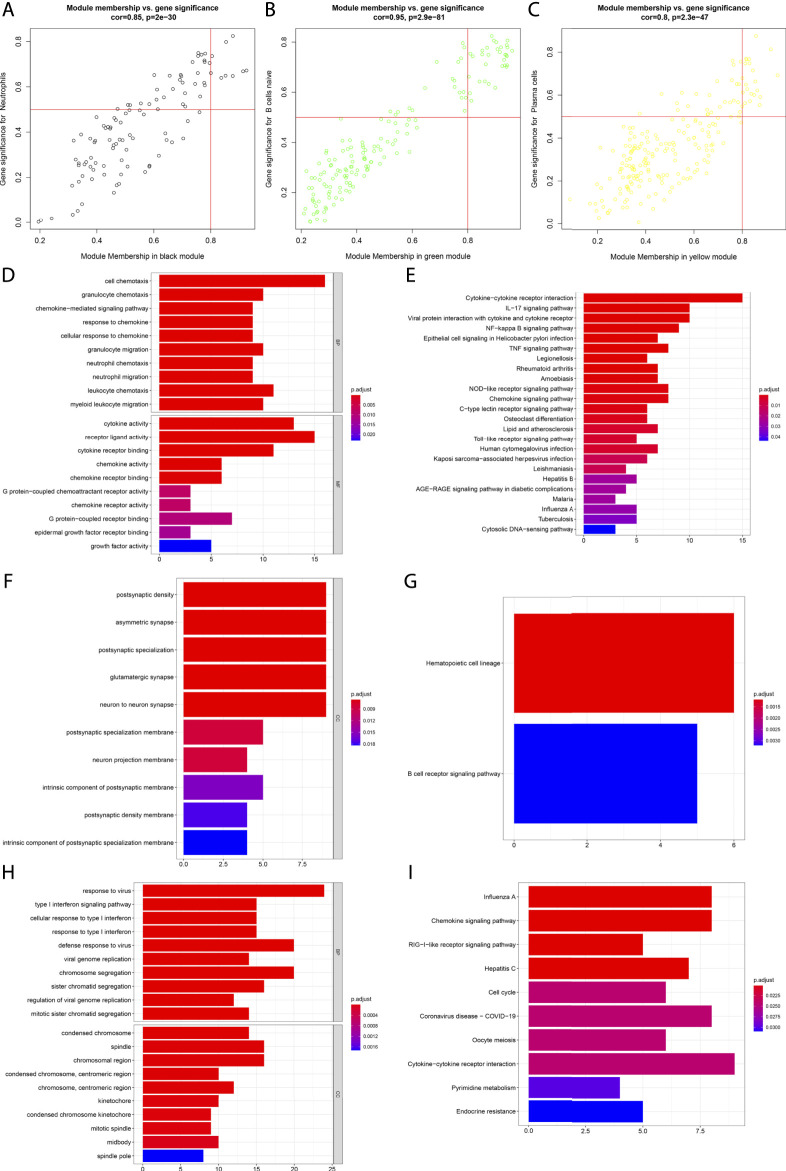
Analyses of immune cell-infiltration-relevant genes and their biological significance. **(A)** Scatter plots depict the interactions of module membership with gene significance for neutrophils in the black module. **(B)** Scatter plots show the interactions of module membership with gene significance for B cells naïve in the green module. **(C)** Scatter plots present the interactions of module membership with gene significance for plasma cells in the yellow module. **(D,E)** GO and KEGG enrichment results of genes in the black module. **(F,G)** GO and KEGG enrichment results of genes in the green module. **(H,I)** GO and KEGG enrichment results of genes in the yellow module.

Scatter analyses demonstrated the remarkable interactions of module membership with gene significance for neutrophils in the black module (Figure 2A). In Figure 2B, there was a prominent correlation between module membership and gene significance for B cells naïve in the green module. Additionally, we noted the significant interactions of module membership with gene significance for plasma cells in the yellow module (Figure 2C). Above data uncovered that the genes in the black, green, and yellow modules presented remarkable correlations to immune cells. Their biological significance was further investigated. It was proven that the genes in the black module were significantly linked to neutrophil chemotaxis and inflammation like cytokine-cytokine receptor interaction, IL-17 and TNF signaling pathways (Figures 2D,E). The genes in the green module presented prominent interactions with hematopoietic cell lineage and B cell receptor signaling pathway (Figures 2F,G). Additionally, the genes in the yellow module were prominently linked to viral infection (Figures 2H,I).

### Determination of Immune Cell-Relevant Hub Genes

**FIGURE 3:**
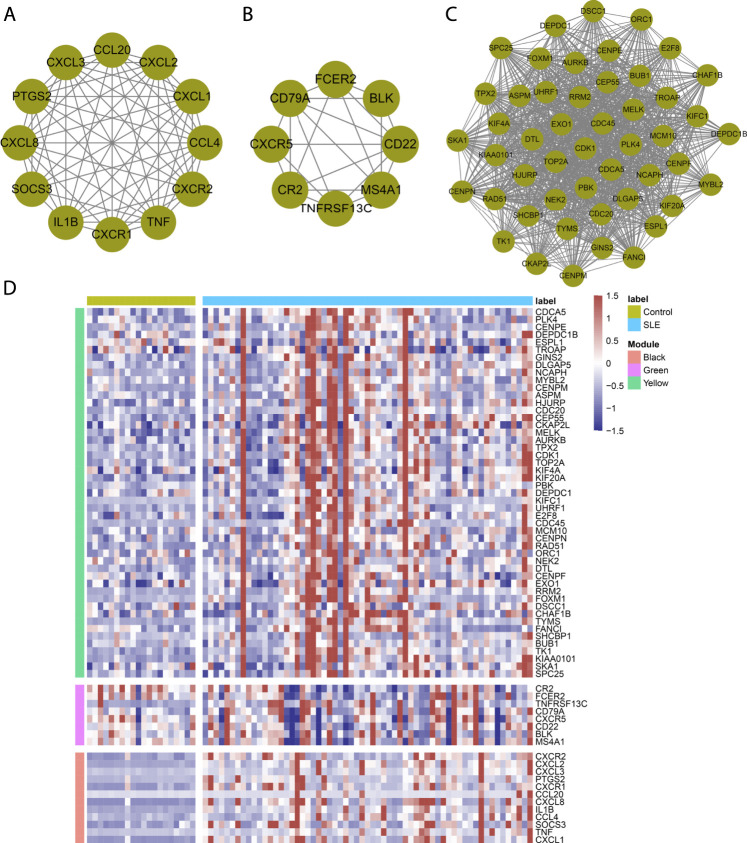
Determination of immune cell-relevant hub genes. **(A–C)** Hub gene clusters in the **(A)** black, **(B)** green, and **(C)** yellow modules through MCODE approach. **(D)** Heatmap depicts the expression of hub genes in SLE and normal PBMCs in the GSE50772 dataset.

With the MCODE approach, hub genes in the immune cell-relevant models were further determined. As a result, there were twelve hub genes (CXCR2, CXCL2, CXCL3, PTGS2, CXCR1, CCL20, CXCL8, IL1B, CCL4, SOCS3, TNF, and CXCL1) in the black model (Figure 3A). Figure 3B depicted eight hub genes (CR2, FCER2, TNFRSF13C, CD79A, CXCR5, CD22, BLK, and MS4A1) in the green model. Additionally, we determined 49 hub genes in the yellow module, containing CDCA5, PLK4, CENPE, DEPDC1B, ESPL1, TROAP, GINS2, DLGAP5, NCAPH, MYBL2, CENPM, ASPM, HJURP, CDC20, CEP55, CKAP2L, MELK, AURKB, TPX2, CDK1, TOP2A, KIF4A, KIF20A, PBK, DEPDC1, KIFC1, UHRF1, E2F8, CDC45, MCM10, CENPN, RAD51, ORC1, NEK2, DTL, CENPF, EXO1, RRM2, FOXM1, DSCC1, CHAF1B, TYMS, FANCI, SHCBP1, BUB1, TK1, KIAA0101, SKA1, and SPC25 (Figure 3C). Figure 3D presented the remarkable discrepancy in hub gene expression between SLE and normal PBMCs in the GSE50772 dataset.

### Establishment of a Least Absolute Shrinkage and Selection Operator Model for Diagnosing Systemic Lupus Erythematosus

**FIGURE 4:**
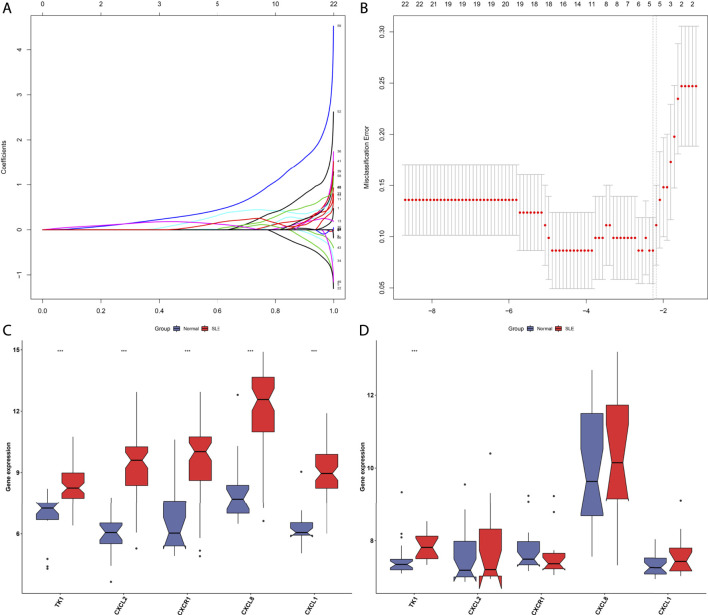
Establishment of a LASSO model for distinguishing SLE from normal controls. **(A)** Ten-fold cross-verification of turning parameter selection in the LASSO model. **(B)** LASSO coefficient profiling of the hub genes. **(C)** Box plots show the expression of characteristic genes in SLE and normal PBMCs in the GSE50772 dataset. **(D)** Verification of the expression of characteristic genes in SLE and normal PBMCs in the GSE81622 dataset. ***p < 0.001.

**FIGURE 5:**
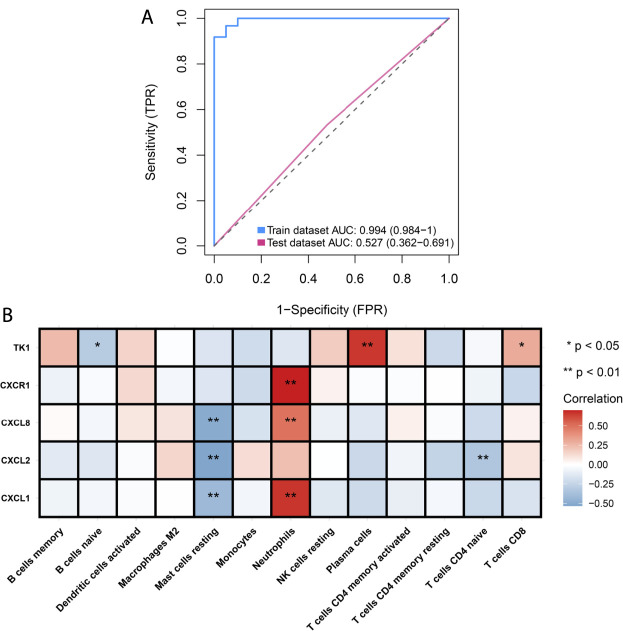
Analyses of diagnostic efficacy of the LASSO model and associations of characteristic genes with infiltrating immune cells. **(A)** ROCs for evaluating the diagnostic efficacy of the LASSO model both in the GSE50772 and GSE81622 datasets. **(B)** Heatmap depicts the associations of characteristic genes (CXCL1, CXCL2, CXCL8, CXCR1 and TK1) with infiltrating immune cells in the GSE50772 dataset. *p < 0.05; **p < 0.01.

Hub genes in the black, green, and yellow modules were integrated for LASSO analyses. Under ten-fold cross-verification, five characteristic genes (CXCL1, CXCL2, CXCL8, CXCR1 and TK1) were determined and the LASSO model was constructed for SLE (Figures 4A,B). All of them presented remarkable up-regulations in SLE compared with normal PBMCs in the GSE50772 dataset (Figure 4C). Their expressions were verified in the GSE81622 dataset. It was proven that TK1 displayed prominent up-regulation in SLE (Figure 4D). ROCs were conducted for estimating the diagnostic potency of this LASSO model. AUC reached 0.994 in the GSE50772 dataset, proving the favorable diagnostic efficacy of this model (Figure 5A). The diagnostic performance was confirmed in the GSE81622 dataset.

### Associations of Characteristic Genes With Infiltrating Immune Cells in Systemic Lupus Erythematosus

Further Spearman correlation analyses were conducted for unraveling the interactions of characteristic genes with infiltrating immune cells. In Figure 5B, CXCL1 and CXCL8 were negatively linked to mast cells resting but were positively correlated with neutrophils. CXCL2 displayed negative interactions with mast cells resting and T cells CD4 naïve. There was a positive interaction of CXCR1 with neutrophils. Additionally, we noted that TK1 showed the negative association with B cells naïve while displayed the positive associations with plasma cells and T cells CD8. These data indicated the tight interactions of characteristic genes with infiltrating immune cells in SLE.

### Signaling Pathways Involving Characteristic Genes

**FIGURE 6:**
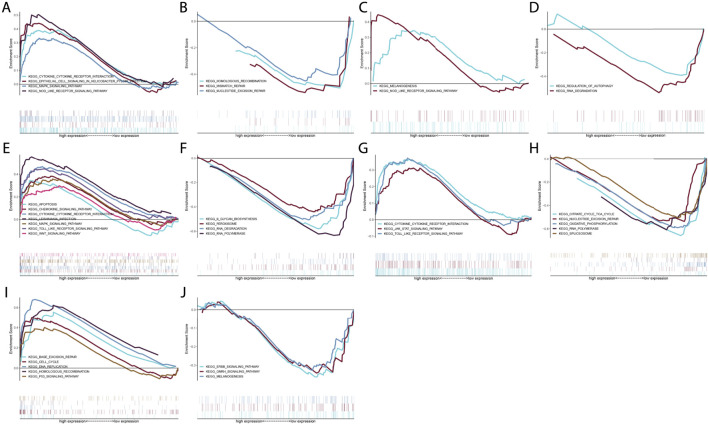
Signaling pathways involving characteristic genes. **(A–J)** GSEA enrichment analyses of high and low expression of characteristic genes: **(A,B)** CXCL1, **(C,D)** CXCL2, **(E,F)** CXCL8, **(G,H)** CXCR1 and **(I,J)** TK1 in the GSE50772 dataset.

GSEA was conducted through comparing high and low expression groups of characteristic genes in the GSE50772 dataset. Our results demonstrated that cytokine-cytokine receptor interaction, epithelial cell signaling in *helicobacter pylori* infection, MAPK signaling pathway and NOD-like receptor signaling pathway were remarkably activated in high CXCL1 expression group (Figure 6A) while homologous recombination, mismatch repair and nucleotide excision repair were prominently activated in low CXCL1 expression group (Figure 6B). CXCL2 presented positive interactions with melanogenesis and NOD-like receptor pathway (Figure 6C) while was negatively linked to regulation of autophagy and RNA degradation (Figure 6D). In Figure 6E, high CXCL8 expression group showed the remarkable activation of apoptosis, chemokine signaling pathway, cytokine-cytokine receptor interaction, leishmania infection, MAPK signaling pathway, TOLL-like receptor signaling pathway and Wnt signaling pathway. But low CXCL8 expression group presented the prominent activation of n-glycan biosynthesis, peroxisome, RNA degradation and RNA polymerase (Figure 6F). We also noted that cytokine-cytokine receptor interaction, JAK-STAT signaling pathway and Toll-like receptor signaling pathway were significantly activated in high CXCR1 expression group (Figure 6G) while citrate cycle TCA cycle, nucleotide excision repair, oxidative phosphorylation, RNA polymerase and spliceosome were remarkably activated in low CXCR1 expression group (Figure 6H). Additionally, base excision repair, cell cycle, DNA replication, homologous recombination as well as p53 pathway presented prominent activation in high TK1 expression group (Figure 6I) while ERBB signaling pathway, GNRH signaling pathway and melanogenesis showed significant activation in low TK1 expression group (Figure 6J). Overall, above data were indicative of the molecular mechanisms underlying characteristic genes.

### Activation of Inflammatory Response in Systemic Lupus Erythematosus Patients

**FIGURE 7:**
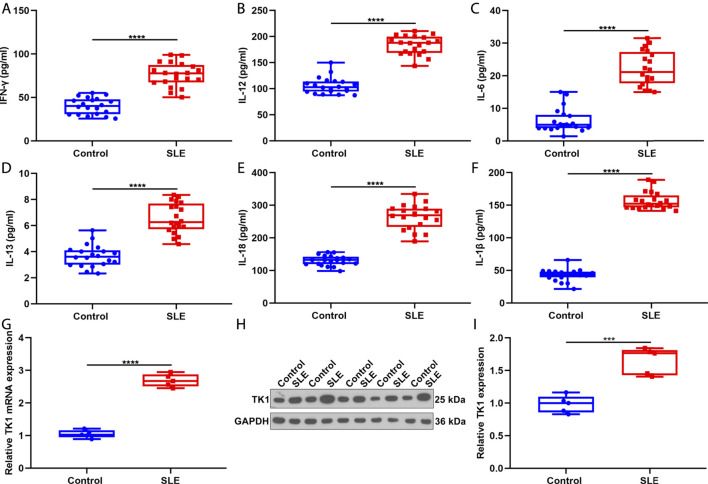
Activation of inflammatory response in SLE and up-regulation of TK1 in SLE BMSCs. **(A–F)** Serum levels of **(A)** IFN-γ, **(B)** IL-12, **(C)** IL-6, **(D)** IL-13, **(E)** IL-18 as well as **(F)** IL-1β were measured in twenty SLE patients and twenty controls through ELISA. **(G–I)** TK1 mRNA expression was tested in BMSCs from five SLE patients and five controls through **(G)** RT-qPCR and **(H,I)** western blotting. ***p < 0.001; ****p < 0.0001.

This study recruited twenty SLE patients and twenty controls and measured the serum levels of inflammatory factors through ELISA. Consequently, IFN-γ, IL-12, IL-6, IL-13, IL-18 and IL-1β presented remarkable up-regulations in serum of SLE patients in comparison to healthy controls (Figures 7A–F). These data proved that inflammatory response was prominently activated in SLE.

### Verification of Up-Regulation of TK1 in Systemic Lupus Erythematosus Bone Marrow Mesenchymal Stem Cells

We isolated BMSCs from five SLE patients and five controls and verified the expression of TK1 in SLE. As expected, our data proved that TK1 mRNA displayed remarkable up-regulation in SLE compared with control BMSCs (Figure 7G). Consistently, TK1 protein was markedly overexpressed in SLE than control BMSCs (Figures 7H,I). Overall, our data proved the up-regulation of TK1 in SLE BMSCs.

### Silencing TK1 Relieves Inflammatory Response and Apoptosis in Systemic Lupus Erythematosus Bone Marrow Mesenchymal Stem Cells

**FIGURE 8:**
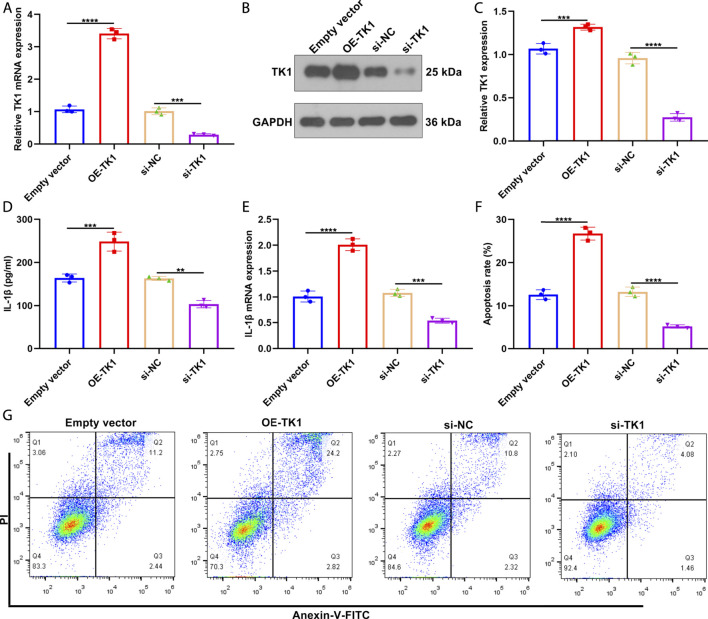
Silencing TK1 relieves inflammatory response and apoptosis in SLE BMSCs. **(A)** TK1 mRNA expression was measured in SLE BMSCs with TK1 overexpression or TK1 knockdown through RT-qPCR. **(B,C)** TK1 protein expression was tested in SLE BMSCs with TK1 overexpression or TK1 knockdown via western blotting. **(D)** IL-1β levels were quantified in the supernatant of specified SLE BMSCs through ELISA. **(E)** RT-qPCR was adopted to measure the mRNA expression of IL-1β in specified SLE BMSCs. **(F,G)** Flow cytometry analyses were conducted to test the apoptosis levels of specified SLE BMSCs. **p < 0.01; ***p < 0.001; ****p < 0.0001.

To investigate the function of TK1 in SLE pathogenesis, TK1 expression was remarkably overexpressed as well as weakened in SLE BMSCs (Figures 8A–C). In Figure 8D, TK1-overexpressed SLE BMSCs presented prominently up-regulated IL-1β levels in the supernatant. Also, decreased IL-1β levels were tested in the supernatant of TK1-silenced SLE BMSCs. We also investigated that TK1 up-regulation elevated IL-1β expression in SLE BMSCs (Figure 8E). Oppositely, IL-1β expression was weakened by TK1 knockdown in SLE BMSCs. Moreover, remarkably enhanced apoptosis levels were noted in TK1-overexpressed SLE BMSCs while the opposite results were investigated in TK1-silenced SLE BMSCs (Figures 8F,G). Overall, targeting TK1 relieved inflammatory response and apoptosis in SLE BMSCs.

### TK1 Knockdown Relieves Apoptosis, Cell Cycle Arrest and Senescence in Systemic Lupus Erythematosus Bone Marrow Mesenchymal Stem Cells

**FIGURE 9:**
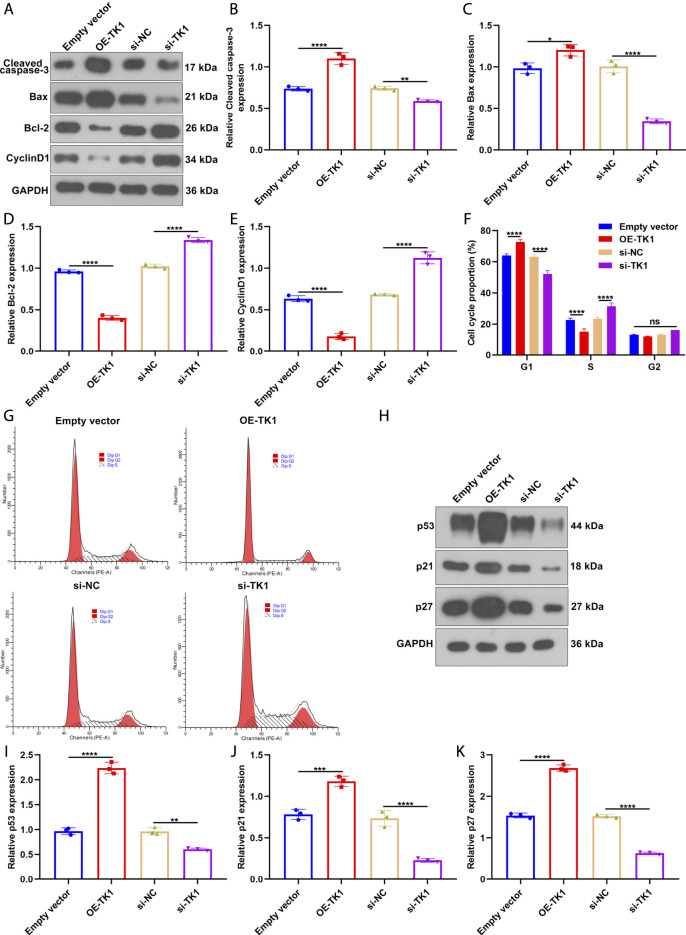
TK1 knockdown relieves apoptosis, cell cycle arrest and senescence in SLE BMSCs. **(A–E)** The expression of cell cycle- and apoptosis-relevant proteins was measured in SLE BMSCs with TK1 overexpression or TK1 knockdown through western blotting, containing **(B)** Cleaved caspase-3, **(C)** Bax, **(D)** Bcl-2 as well as **(E)** CyclinD1. **(F,G)** Cell cycle proportions were tested in specified SLE BMSCs via flow cytometry analyses. **(H–K)** The expression of senescence-relevant proteins was measured in specified SLE BMSCs. Ns: not significant; *p < 0.05; **p < 0.01; ***p < 0.001; ****p < 0.0001.

Further, we measured the influence of TK1 on the expression of cell cycle- and apoptosis-relevant proteins in SLE BMSCs via western blotting (Figure 9A). As a result, TK1 up-regulation facilitated Cleaved caspase-3 (Figure 9B) and Bax (Figure 9C) expressions but weakened the expression of Bcl-2 (Figure 9D) in SLE BMSCs. Also, the expression of Cleaved caspase-3 an Bax was reduced as well as the expression of Bcl-2 was enhanced in TK1-silenced SLE BMSCs. Hence, silencing TK1 relieved apoptosis of SLE BMSCs. Also, cell cycle-relevant protein CyclinD1 expression was remarkably reduced in TK1-overexpressed SLE BMSCs as well as it was enhanced in TK1-silenced SLE BMSCs (Figure 9E). In Figures 9F,G, G1 arrest was investigated in TK1-overexpressed SLE BMSCs. Oppositely, G1 phase was remarkably shortened by TK1 knockdown in SLE BMSCs. The influence of TK1 on senescence was further investigated in SLE BMSCs. Consequently, there was prominently enhanced expression of senescence-relevant proteins containing p53, p21 and p27 in TK1-overexpressed SLE BMSCs (Figures 9H–K). Also, their expression was weakened in TK1-silenced SLE BMSCs. Overall, TK1 knockdown relieved apoptosis, cell cycle arrest and senescence in SLE BMSCs.

## Discussion

SLE represents a chronic autoimmune rheumatic disease with high heterogeneity in clinical presentations, treatment responses and clinical outcomes (Arnaud and Tektonidou 2020). Due to the highly complicated pathogenesis of SLE, to comprehend pathophysiology provides an in-depth understanding of the molecular mechanisms (Arnaud and Tektonidou 2020). The activity of SLE severely depends on evaluating inflammation in multiple organs (Fousert et al., 2020; Wei et al., 2020). Computational and biological, advances in bioinformatics accelerate the capacity of predicting alterations in SLE activity as well as optimizing treatment modalities (Banchereau et al., 2016; Yang et al., 2020; Zhao et al., 2021).

SLE is characterized by autoimmune, and inflammatory processes (Zhao et al., 2021). Our CIBERSORT results demonstrated the widespread infiltration of diverse immune cell populations in SLE, consistent with previous research (Zhao et al., 2021). The experimental evidences showed the remarkable up-regulations of serum levels of inflammatory chemokines containing IFN-γ, IL-12, IL-6, IL-13, IL-18 and IL-1β in SLE patients than controls, proving the activation of inflammatory response in SLE. With WGCNA approach, we determined three immune cell-relevant co-expression modules that displayed strong correlations to neutrophils, B cells naïve, and plasma cells. Further functional enrichment analyses proved the crucial roles of genes in the three modules in modulating neutrophils, B cells naïve, and plasma cells. Thus, above genes could be utilized as immune cell-relevant genes. With MCODE approach, immune cell-relevant hub genes were determined. Among them, five characteristic genes were screened (containing CXCL1, CXCL2, CXCL8, CXCR1 and TK1) with LASSO approach. These characteristic genes displayed remarkable up-regulations in SLE in comparison to controls. The AUC of ROCs was 0.994 in the GSE50772 dataset, proving the favorable diagnostic efficacy of this multivariate model. Further analyses were indicative of the interactions of five characteristic genes with diverse immune cells in SLE. Additionally, GSEA results demonstrated that five characteristic genes exerted crucial roles in modulating distinct pathways. Chemokines exert remarkable functions in the pathogenesis of SLE, not only inducing autoimmune response in multiple organs, but also amplifying the induced inflammatory response (Ghafouri-Fard et al., 2021). For instance, decreasing CXCL2 facilitates innate immune activation and neutrophil extracellular trap formation (Yang et al., 2021). Nevertheless, the roles of TK1 in SLE progression remain indistinct.

BMSCs have been proposed as promising and alternative cells for treatment of SLE due to the self-renewal, pluripotent differentiation capacity and reduced immunogenicity (Gao et al., 2020). Previous research indicated allogenic BMSC transplantation as a safe and effective treatment against refractory SLE (Sun et al., 2009). Unfortunately, in-depth research proved that syngeneic BMSC transplantation is not effective (Gu et al., 2012). Recent evidences suggest that SLE BMSCs characterized by apoptosis, cell cycle arrest, and senescent phenotype could result in SLE progression (Ji et al., 2019). Here, we isolated BMSCs from five SLE patients and five controls. TK1 expression was proved to be up-regulated in BMSCs of SLE. TK1-overexpressed BMSCs of SLE presented enhanced IL-1β expression, apoptosis, G1 arrest and senescent phenotype as well as the opposite results were proved in TK1-silenced BMSCs of SLE. Hence, our findings might offer a novel therapeutic agent against SLE. Despite this, there are several limitations in our study. Firstly, the number of SLE patients is limited, and more patients should be included to validate the expression of TK1 in BMSCs of SLE patients. Secondly, *in vivo* experiments are required to verify the roles of TK1 in BMSCs of SLE. Thirdly, the molecular mechanisms of TK1 in modulating BMSCs of SLE should be further explored.

## Conclusion

Collectively, our study demonstrated that TK1 triggered inflammatory response, apoptosis, cell cycle arrest and senescence in BMSCs of SLE and suppressing TK1 remarkably alleviated inflammation, growth arrest and senescent phenotype of SLE BMSCs. Hence, TK1 blockade might become a potential therapeutic target against SLE. In our future studies, we will validate the therapeutic roles of TK1-silenced BMSCs against SLE through *in vivo* experiments.

## Data Availability

The original contributions presented in the study are included in the article/supplementary material, further inquiries can be directed to the corresponding author.

## Glossary

AUC: area under curve
BMSCs: bone marrow mesenchymal stem cells
CIBERSORT: cell-type identification by estimating relative subsets of RNA transcript
ELISA: Enzyme-linked immunosorbent assay
GEO: Gene Expression Omnibus
GO: Gene ontology
GS: gene significance
GSEA: Gene set enrichment analyses
IFN-γ: interferon-γ
IL-12: interleukin-12
IL-13: interleukin-13
IL-18: interleukin-18
IL-1β: interleukin-1β
IL-6: interleukin-6
KEGG: Kyoto encyclopedia of genes and genomes
LASSO: Least absolute shrinkage and selection operator
MCODE: Molecular Complex Detection
MEs: module eigengenes
MM: module membership
PBMCs: peripheral blood mononuclear cells
PPI: protein-protein interaction
ROC: receiver operator characteristic curve
RT-qPCR: real-time quantitative polymerase chain reaction
SLE: systemic lupus erythematosus
STRING: Search Tool for Retrieval of Interacting Genes/Proteins
TOM: topological overlap matrix
WGCNA: weighted gene co-expression analyses

## References

[B1] Arnaud L. Tektonidou M. G. (2020). Long-term Outcomes in Systemic Lupus Erythematosus: Trends over Time and Major Contributors. Rheumatology (Oxford) 59 (Suppl. 5), v29–v38. 10.1093/rheumatology/keaa382 33280012 PMC7719040

[B2] Banchereau R. Hong S. Cantarel B. Baldwin N. Baisch J. Edens M. (2016). Personalized Immunomonitoring Uncovers Molecular Networks that Stratify Lupus Patients. Cell 165 (3), 551–565. 10.1016/j.cell.2016.03.008 27040498 PMC5426482

[B3] Doncheva N. T. Morris J. H. Gorodkin J. Jensen L. J. (2019). Cytoscape StringApp: Network Analysis and Visualization of Proteomics Data. J. Proteome Res. 18 (2), 623–632. 10.1021/acs.jproteome.8b00702 30450911 PMC6800166

[B4] Durcan L. O'Dwyer T. Petri M. (2019). Management Strategies and Future Directions for Systemic Lupus Erythematosus in Adults. The Lancet 393 (10188), 2332–2343. 10.1016/s0140-6736(19)30237-5 31180030

[B5] Engebretsen S. Bohlin J. (2019). Statistical Predictions with Glmnet. Clin. Epigenet 11 (1), 123. 10.1186/s13148-019-0730-1 PMC6708235 31443682

[B6] Fousert E. Toes R. Desai J. (2020). Neutrophil Extracellular Traps (NETs) Take the Central Stage in Driving Autoimmune Responses. Cells 9 (4), 915. 10.3390/cells9040915 PMC7226846 32276504

[B7] Furie R. Werth V. P. Merola J. F. Stevenson L. Reynolds T. L. Naik H. (2019). Monoclonal Antibody Targeting BDCA2 Ameliorates Skin Lesions in Systemic Lupus Erythematosus. J. Clin. Invest. 129 (3), 1359–1371. 10.1172/jci124466 30645203 PMC6391094

[B8] Gao L. OConnell M. Liesveld J. McDavid A. Anolik J. H. Looney R. J. (2020). Bone Marrow Mesenchymal Stem Cells from Patients with SLE Maintain an Interferon Signature during *In Vitro* Culture. Cytokine 132, 154725. 10.1016/j.cyto.2019.05.012 31153744

[B9] Geng L. Tang X. Wang S. Sun Y. Wang D. Tsao B. P. (2020). Reduced Let-7f in Bone Marrow-Derived Mesenchymal Stem Cells Triggers Treg/Th17 Imbalance in Patients with Systemic Lupus Erythematosus. Front. Immunol. 11, 233. 10.3389/fimmu.2020.00233 32133007 PMC7040072

[B10] Geng L. Tang X. Zhou K. Wang D. Wang S. Yao G. (2019). MicroRNA-663 Induces Immune Dysregulation by Inhibiting TGF-Β1 Production in Bone Marrow-Derived Mesenchymal Stem Cells in Patients with Systemic Lupus Erythematosus. Cell Mol Immunol 16 (3), 260–274. 10.1038/cmi.2018.1 30886422 PMC6460486

[B11] Ghafouri-Fard S. Shahir M. Taheri M. Salimi A. (2021). A Review on the Role of Chemokines in the Pathogenesis of Systemic Lupus Erythematosus. Cytokine 146, 155640. 10.1016/j.cyto.2021.155640 34252872

[B12] Gu F. Molano I. Ruiz P. Sun L. Gilkeson G. S. (2012). Differential Effect of Allogeneic versus Syngeneic Mesenchymal Stem Cell Transplantation in MRL/lpr and (NZB/NZW)F1 Mice. Clin. Immunol. 145 (2), 142–152. 10.1016/j.clim.2012.08.012 23041504

[B13] Hanaoka H. Nishimoto T. Okazaki Y. Takeuchi T. Kuwana M. (2020). A Unique Thymus-Derived Regulatory T Cell Subset Associated with Systemic Lupus Erythematosus. Arthritis Res. Ther. 22 (1), 88. 10.1186/s13075-020-02183-2 32317002 PMC7171795

[B14] Ji J. Fu T. Dong C. Zhu W. Yang J. Kong X. (2019). Targeting HMGB1 by Ethyl Pyruvate Ameliorates Systemic Lupus Erythematosus and Reverses the Senescent Phenotype of Bone Marrow-Mesenchymal Stem Cells. Aging 11 (13), 4338–4353. 10.18632/aging.102052 31303606 PMC6660056

[B15] Kennedy W. P. Maciuca R. Wolslegel K. Tew W. Abbas A. R. Chaivorapol C. (2015). Association of the Interferon Signature Metric with Serological Disease Manifestations but Not Global Activity Scores in Multiple Cohorts of Patients with SLE. Lupus Sci. Med. 2 (1), e000080. 10.1136/lupus-2014-000080 25861459 PMC4379884

[B16] Khodoun M. Chimote A. A. Ilyas F. Z. Duncan H. J. Moncrieffe H. Kant K. S. (2020). Targeted Knockdown of Kv1.3 Channels in T Lymphocytes Corrects the Disease Manifestations Associated with Systemic Lupus Erythematosus. Sci. Adv. 6 (47), eabd1471. 10.1126/sciadv.abd1471 33208373 PMC7673800

[B17] Kiriakidou M. Ching C. L. (2020). Systemic Lupus Erythematosus. Ann. Intern. Med. 172 (11), Itc81–itc96. 10.7326/aitc202006020 32479157

[B18] Langfelder P. Horvath S. (2008). WGCNA: an R Package for Weighted Correlation Network Analysis. BMC Bioinformatics 9, 559. 10.1186/1471-2105-9-559 19114008 PMC2631488

[B19] Liberzon A. Birger C. Thorvaldsdóttir H. Ghandi M. Mesirov J. P. Tamayo P. (2015). The Molecular Signatures Database Hallmark Gene Set Collection. Cel Syst. 1 (6), 417–425. 10.1016/j.cels.2015.12.004 PMC4707969 26771021

[B20] Mathias L. M. Stohl W. (2020). Systemic Lupus Erythematosus (SLE): Emerging Therapeutic Targets. Expert Opin. Ther. Targets 24 (12), 1283–1302. 10.1080/14728222.2020.1832464 33034541

[B21] Newman A. M. Liu C. L. Green M. R. Gentles A. J. Feng W. Xu Y. (2015). Robust Enumeration of Cell Subsets from Tissue Expression Profiles. Nat. Methods 12 (5), 453–457. 10.1038/nmeth.3337 25822800 PMC4739640

[B22] Robin X. Turck N. Hainard A. Tiberti N. Lisacek F. Sanchez J.-C. (2011). pROC: an Open-Source Package for R and S+ to Analyze and Compare ROC Curves. BMC Bioinformatics 12, 77. 10.1186/1471-2105-12-77 21414208 PMC3068975

[B23] Subramanian A. Tamayo P. Mootha V. K. Mukherjee S. Ebert B. L. Gillette M. A. (2005). Gene Set Enrichment Analysis: a Knowledge-Based Approach for Interpreting Genome-wide Expression Profiles. Proc. Natl. Acad. Sci. 102 (43), 15545–15550. 10.1073/pnas.0506580102 16199517 PMC1239896

[B24] Sun L. Akiyama K. Zhang H. Yamaza T. Hou Y. Zhao S. (2009). Mesenchymal Stem Cell Transplantation Reverses Multiorgan Dysfunction in Systemic Lupus Erythematosus Mice and Humans. Stem Cells 27 (6), 1421–1432. 10.1002/stem.68 19489103 PMC2704254

[B25] Szklarczyk D. Morris J. H. Cook H. Kuhn M. Wyder S. Simonovic M. (2017). The STRING Database in 2017: Quality-Controlled Protein-Protein Association Networks, Made Broadly Accessible. Nucleic Acids Res. 45 (D1), D362–d368. 10.1093/nar/gkw937 27924014 PMC5210637

[B26] Tan W. Gu Z. Leng J. Zou X. Chen H. Min F. (2019). Let-7f-5p Ameliorates Inflammation by Targeting NLRP3 in Bone Marrow-Derived Mesenchymal Stem Cells in Patients with Systemic Lupus Erythematosus. Biomed. Pharmacother. 118, 109313. 10.1016/j.biopha.2019.109313 31545233

[B27] Wei K. Korsunsky I. Korsunsky I. Marshall J. L. Gao A. Watts G. F. M. (2020). Notch Signalling Drives Synovial Fibroblast Identity and Arthritis Pathology. Nature 582 (7811), 259–264. 10.1038/s41586-020-2222-z 32499639 PMC7841716

[B28] Yang B. Huang X. Xu S. Li L. Wu W. Dai Y. (2021). Decreased miR-4512 Levels in Monocytes and Macrophages of Individuals with Systemic Lupus Erythematosus Contribute to Innate Immune Activation and Neutrsophil NETosis by Targeting TLR4 and CXCL2. Front. Immunol. 12, 756825. 10.3389/fimmu.2021.756825 34721432 PMC8552026

[B29] Yang F. Zhai Z. Luo X. Luo G. Zhuang L. Zhang Y. (2020). Bioinformatics Identification of Key Candidate Genes and Pathways Associated with Systemic Lupus Erythematosus. Clin. Rheumatol. 39 (2), 425–434. 10.1007/s10067-019-04751-7 31673979

[B30] Yang J. Yang X. Zou H. Chu Y. Li M. (2011). Recovery of the Immune Balance between Th17 and Regulatory T Cells as a Treatment for Systemic Lupus Erythematosus. Rheumatology 50 (8), 1366–1372. 10.1093/rheumatology/ker116 21489974

[B31] Yu G. Wang L.-G. Han Y. He Q.-Y. (2012). clusterProfiler: an R Package for Comparing Biological Themes Among Gene Clusters. OMICS: A J. Integr. Biol. 16 (5), 284–287. 10.1089/omi.2011.0118 PMC3339379 22455463

[B32] Yuan X. Qin X. Wang D. Zhang Z. Tang X. Gao X. (2019). Mesenchymal Stem Cell Therapy Induces FLT3L and CD1c+ Dendritic Cells in Systemic Lupus Erythematosus Patients. Nat. Commun. 10 (1), 2498. 10.1038/s41467-019-10491-8 31175312 PMC6555800

[B33] Zhao X. Zhang L. Wang J. Zhang M. Song Z. Ni B. (2021). Identification of Key Biomarkers and Immune Infiltration in Systemic Lupus Erythematosus by Integrated Bioinformatics Analysis. J. Transl Med. 19 (1), 35. 10.1186/s12967-020-02698-x 33468161 PMC7814551

[B34] Zhu H. Mi W. Luo H. Chen T. Liu S. Raman I. (2016). Whole-genome Transcription and DNA Methylation Analysis of Peripheral Blood Mononuclear Cells Identified Aberrant Gene Regulation Pathways in Systemic Lupus Erythematosus. Arthritis Res. Ther. 18, 162. 10.1186/s13075-016-1050-x 27412348 PMC4942934

